# Acculturative stress and mental health among international students in Germany: the protective roles of mindfulness, optimism, self-efficacy and coping strategies

**DOI:** 10.1186/s40359-026-04576-5

**Published:** 2026-04-25

**Authors:** Constance Karing, Erhabor S. Idemudia

**Affiliations:** 1 Catholic University of Eichstätt-Ingolstadt, Ostenstraße 25, Eichstätt, 85072 Germany; 2 Friedrich-Schiller-University Jena, Jena , Germany; 3 North-West University, Mmabatho, South Africa

**Keywords:** International students, Mental health, Suicide, Acculturative stress, Mindfulness, Coping

## Abstract

**Background:**

Germany is a popular destination for international students. However, little is known about their mental health issues. Further, there is limited research in European countries investigating risk and protective factors for acculturative stress and mental health issues among international student samples. This study aimed to investigate the prevalence of depression, anxiety and acculturative stress among international students in Germany. Further, we examined the association of possible protective and risk factors with these outcome variables.

**Methods:**

A total of 327 international students in Germany completed an online survey. Standardised measures for depression (PHQ-9), anxiety (GAD-7), and acculturative stress were used. Hierarchical regression analyses were employed to assess the impact of demographic factors, psychological variables, and coping strategies on mental health outcomes.

**Results:**

The prevalence of depression and anxiety was high among international students (46.5% and 46.8%, respectively). A substantial proportion of the sample (31.2%) reported suicidal ideation or thoughts of self-harm. Further, international students in our study reported moderate acculturative stress. Mindfulness, different sources of social support, self-efficacy, optimism and acceptance were protective factors against depression, anxiety or acculturative stress. However, less than half of our sample was well supported by university facilities. A few demographic variables (gender, graduation, education, home country) were related to higher acculturative stress and anxiety. Acculturative stress was a significant predictor of depression and anxiety.

**Conclusion:**

Our findings highlight the importance of addressing mental health issues among international students. The results suggest that universities may consider providing adequate psychological services and strengthening institutional support for international students.

## Introduction

Over 6.4 million international students are pursuing higher education abroad, with significant numbers enrolled in countries such as the United States, the United Kingdom, Australia, Germany, Canada, France, and China [1]. In Germany, international student enrolment reached 379.900 in the winter semester of 2023/2024, accounting for 13% of all students in the country [2]. These students predominantly come from Asia - particularly India and China - and Europe, including countries like Austria, Italy, and Ukraine [2].

International students often face a multitude of challenges that can hinder their academic success, social life and well-being. Language barriers, homesickness, financial difficulties, and the need to adjust to a new academic system and culture are common obstacles [3–5]. These difficulties can lead to experiences of acculturative stress and mental health issues, increasing the risk of lower academic achievement and university drop-out [6–9].

Acculturative stress refers to the effects of various psychological and behavioral stressors that individuals (e.g., international students) experience when living in another culture [10, 11]. Berry [10, 11] conceptualises acculturative stress based on the transactional model of stress and coping [12, 13]. According to Berry’s model of acculturative stress [11], if an international student evaluates the challenges resulting from intercultural contact as a threat, and doubts that he can manage these challenges, he can experience acculturative stress. The longer the stress endured, the higher the risk for mental health issues (e.g., depression, anxiety) and poorer sociocultural adaptation [11, 14]. Numerous studies on international students’ acculturative stress have reported that acculturative stress consists of several dimensions [15], such as language barriers (e.g., difficulty in communicating effectively in an academic or social setting), cultural dissonance (e.g., struggles with cultural norms, expectations, and values that differ from local society), social isolation (e.g., limited social networks, difficulty forming connections) and guilt (e.g., feeling guilty to leave family and friends behind) [15–17]. Consistent with the acculturative stress theory [11], a meta-analysis by Soufi Amlashi et al. [14] showed a moderate positive correlation between acculturative stress and several negative psychological outcomes in international students (e.g., mental health symptoms, negative affect, low life satisfaction, low quality of life, drinking behaviors). Other studies found that acculturative stress was associated with lower social and academic integration [18, 19]. Another meta-analysis reported a positive association between acculturative stress and depression among international students, indicating a large effect [20]. High rates of depression and anxiety have been documented among international students in various countries [7, 9, 21, 22]. For instance, Koppenborg et al. [21] found in a pre-pandemic study with international students in the Netherlands that 33.7% showed a clinically relevant depression and 32.6% reported a clinically relevant anxiety. Another study showed even higher rates of depression and anxiety amongst international students in Lithuania (59% depression, 36% anxiety), but this was at the beginning of the COVID-19 pandemic [22].

## Variables related to international students’ mental health outcomes

Several studies have investigated the predictors of acculturative stress and mental health issues among international students. Most of these studies have been done in the US and Asia, while only a few were done in European countries [23]. Germany represents a unique context for international students due to its low or non-existent tuition fees, comparatively high proportion of international enrolment, and specific linguistic and bureaucratic demands [2]. In contrast to classical destination countries such as the United States or the United Kingdom, international students in Germany often encounter structural challenges related to language requirements, administrative procedures, and limited access to the labour market [2]. These context-specific demands may increase the complexity of adaptation processes and contribute to heightened psychological strain, potentially differing from the English-speaking contexts that dominate existing research. As a result, previously reported associations between coping strategies, acculturative stress, and mental health may not fully generalise to this structurally more demanding context [24, 25].

Prior research on the association between demographic factors (e.g., age, gender, graduation, continent of origin) and acculturative stress has reported mixed results. Some studies found no significant associations with acculturative stress [26–28], while others reported higher stress among younger, male, or postgraduate students [29, 30]. Similarly, female students often report higher depressive and anxious symptoms, though findings are inconsistent [31–34]. Residence duration and continent of origin have also shown mixed effects on acculturative stress and no effects on mental health [17, 26, 30, 35–37]. Altogether, these findings suggest that demographic factors alone are insufficient to explain variation in acculturative stress and mental health outcomes, highlighting the need to consider additional psychological variables.

Internal psychological variables like mindfulness, optimism, self-efficacy, and effective coping strategies may mitigate acculturative stress and enhance mental health among international students [24, 25]. These can be understood through resilience and cultural adaptation frameworks [11, 38, 39]. Resilience involves positive adaptation despite adversity, relying on internal (e.g., self-efficacy, optimism, mindfulness) and contextual (e.g., social support) resources [38]. In the context of studying abroad, these internal and contextual resources may facilitate adaptive responses to acculturative challenges. From a cultural adaptation perspective [10, 11, 39], these resources may support both psychological adjustment (e.g., mental health) and sociocultural adaptation (e.g., effective functioning in the host culture). However, research is limited and mixed: self-efficacy and optimism show protective effects in some studies [6, 41, 42], but not consistently [6, 95]. Research on mindfulness is also scarce with mixed findings [21, 44].

Previous research on coping has shown the importance of perceived control [45, 46]. Rothbaum et al. [47] differentiated between primary (or problem-focused) and secondary control strategies. Primary control strategies (e.g., active coping, planning, seeking social support) refer to attempts to change one’s stressful situation. In contrast, secondary control strategies (e.g., acceptance) are attempts to adjust to the situation psychologically [48, 49]. Secondary coping is often more effective in uncontrollable situations, such as studying abroad [49, 50]. Szabo et al. [49] found that primary coping (active coping, planning) increased anxiety in international students. Social support, however, generally reduces acculturative stress [17, 51–54] and is linked to lower depressive and anxious symptoms [33]. Other research showed that international students use different social support sources [52, 53]. Several studies found that higher levels of social support from host locals or host national students were related to lower levels of acculturative stress [17, 54]. Conversely, insufficient financial support can increase stress and mental health issues among international students [17, 32, 33].

### Integration and theoretical framing

Although prior research has identified multiple predictors of acculturative stress and mental health, these factors are often examined in isolation. Drawing on the transactional model of stress and coping and acculturative stress theory [11, 13], the present study integrates demographic characteristics, psychological resources, and coping strategies within a single framework to examine their relative contributions.

From a theoretical perspective [13, 56], stress responses are shaped by cognitive appraisals of situational demands and perceived coping resources. In the context of studying abroad, acculturative challenges may be appraised as threatening when individuals perceive them as exceeding their coping capacities, increasing the risk of stress and mental health issues. Internal psychological resources such as mindfulness, optimism, and self-efficacy are expected to influence these appraisal processes. For example, mindfulness may reduce the perceived threat of stressors by fostering present-moment awareness and non-judgmental acceptance [44, 57, 58], which is particularly relevant in situations that are difficult to control. Whereas optimism and self-efficacy may enhance individuals’ perceived ability to cope with acculturative demands, thereby reducing stress responses [41, 59]. Coping strategies further shape stress outcomes depending on whether individuals attempt to change or adapt to stressors [13, 48, 49]. Within this integrated framework, acculturative stress is conceptualised as a proximal outcome and key predictor of mental health outcomes.

## Objectives

While Germany is a popular destination for international students, most studies on risk and protective factors for acculturative stress and mental health have been conducted in the United States and Asian countries, which differ substantially from European higher education systems in terms of funding structures, cultural diversity, and integration policies [23]. These differences limit the generalisability of previous findings to the German context. Moreover, European studies remain scarce and have yielded mixed results [21, 30, 94], highlighting the need for context-specific research.

The present study, therefore aims to extend existing research by examining acculturative stress and mental health among international students in Germany, a context that has been largely understudied. Specifically, we investigate (1) the prevalence of mental health issues (anxiety and depression) and acculturative stress among international students in Germany. Based on previous findings [7, 22, 29, 59], we assumed high rates of anxiety and depression and acculturative stress among international students in Germany. Further, we investigate (2) the association of potential protective and risk factors with acculturative stress, depression and anxiety. Based on the transactional model of stress and coping [13] and prior research on international students [17, 29, 32], we expected that internal psychological resources (self-efficacy, optimism, and mindfulness) and coping strategies (acceptance, problem-solving, and social support) would be negatively associated with acculturative stress and mental health issues (anxiety and depression). Furthermore, consistent with the transactional model of stress and coping and resilience theory [11, 13, 38], and the distinction between primary and secondary control strategies [48, 49], we expected that acceptance-based processes, including mindfulness and secondary coping (acceptance), would show the strongest negative associations with acculturative stress and mental health issues, as they are particularly adaptive in situations that are difficult to control.

By integrating these variables within a single framework, the study seeks to provide a more comprehensive understanding of risk and protective factors for international students in a European context.

## Method

### Participants and study design

Three hundred and twenty-seven international students in Germany completed the survey and were included in the present study. A prior sample size analysis for multiple regression was conducted in G*Power 3.1 [60] to determine a sufficient sample size using a power of 0.80, a small effect size (f^2^ = 0.02), and α = 0.05. The results indicated a sufficient sample size of 311 international students. An online cross-sectional design was used. University students were recruited using convenience and snowball sampling techniques. Participants were recruited through university mailing lists and social media platforms targeting international student groups. Inclusion criteria were: (a) being an international student enrolled in a German university and (b) being aged 18 years or older. An invitation link to the online survey was sent to the students in the summer of 2023. The survey included an informed consent page which all international students had to accept before answering the survey questions. The study was approved by the institutional review board at the University of Jena and in accordance with the ethical standards of the 1964 Helsinki Declaration and its later amendments or comparable ethical standards.

## Measures

Both the outcome variables and the independent variables were measured with validated instruments that are used in research with university students.

### Outcome variables

Depression was measured with the Patient Health Questionnaire-9 scale (PHQ-9) [61]. The scale consists of nine items, and responses were scored on a 4-point Likert scale (0 = not at all; 3 = nearly every day). The total score ranges from 0 to 27. The severity of depression was categorised as follows: no/minimal (0–4), mild (5–9), moderate (10–14), serious (15–19) and very serious depression (20–27) [61]. An example item was “Feeling down, depressed, or hopeless”. Cronbach’s α was 0.89.

Anxiety was measured using the 7-item Generalized Anxiety Disorder scale (GAD-7) [62, 63]. Participants rated items like “Feeling nervous, anxious, or on edge” on a 4-point Likert scale (0 = not at all; 3 = nearly every day). The total score ranges between 0 and 21. The severity of anxiety was categorised as follows: no/minimal (0–4), mild (5–9), moderate (10–14) and severe anxiety (15–21) [63]. Cronbach’s α was 0.89.

Acculturative Stress was assessed with the 36-item Acculturative Stress Scale for International Students [16]. Participants responded on a 5-point Likert scale (1 = strongly disagree; 5 = strongly agree) to items like “Homesickness bothers me.” and “I feel insecure here.” Higher scores indicated higher acculturative stress. Cronbach’s α was 0.95.

### Predictors

Demographics University students were asked to state their gender, age, home country, graduation date, educational background of their parents, and time spent in Germany (see Table 1).

**Table 1:** Descriptive statistics of the investigated variables

	Mean (SD)/ *N*(frequency)
Demographics	
Age	26.21 (4.07)
Gender (female)	201 (61.5%)
Graduation	
Undergraduate	74 (22.6%)
Graduate	185 (56.6%)
Postgraduate	68 (20.8%)
Length of stay in Germany (months)	29.14 (26.14)
Continent of origin	
Europe	63 (19.3%)
Africa	22 (6.7%)
Asia	203 (62.1%)
North America	14 (4.3%)
South America	23 (7.0%)
Parents education	
No or primary education	13 (4%)
Secondary education	79 (24.1%)
Higher education	235 (71.9%)
Psychological Variables	
Optimism	4.72 (1.12)
Self-efficacy	4.23 (1.00)
Mindfulness	3.64 (0.94)
Coping	
Problem solving	3.81 (0.79)
Acceptance	3.33 (0.83)
Social support with family	5.06 (1.49)
Social support with friends	5.24 (1.42)
Social support from fellow students	3.33 (1.13)
Institutional support	3.37 (0.92)
Source of financial support	
Parents	167 (51.1%)
Scholarship	73 (22.3%)
Student loan and education fund	24 (7.3%)
Job	177 (54.1%)
Outcome variables	
Depression	10.27 (6.93)
Anxiety	9.39 (5.50)
Acculturative stress	2.42 (0.74)

### Mindfulness

 The Mindfulness Attention and Awareness Scale (MAAS) [43] was used to measure dispositional mindfulness. It is a 15-item scale (1 = *almost never*; 6 = *almost always*, „I rush through activities without being really attentive to them”). The MAAS scale measures the frequency of mindlessness [57]. Thus, the answers were reverse-coded to assess mindfulness. Higher scores on the scale indicated greater mindfulness. Cronbach’s α was 0.90.

### Optimism and self-efficacy

For this study, the subscales of optimism and self-efficacy from the Compound Psychological Capital Scale [64] were used to assess optimism and self-efficacy. Three items each measured the subscales (1 = *strongly disagree*; 6 = *strongly agree;* „The future holds a lot of good in store for me.” [optimism], „I am confident that I could deal efficiently with unexpected events.” [self-efficacy]). Higher scores indicated higher optimism and self-efficacy. Cronbach’s α for optimism was 0.87 and for self-efficacy 0.79.

### Coping strategies

Coping strategies were measured using the subscales problem solving (“I think about possible solutions for how to change the situation.”, 4 items, α =.82) and acceptance (e.g. „When I cannot change something, I accept the situation as it is.”, 3 items, α = 0.75) from the Heidelberg Form for Emotion Regulation Strategies (HFERST) [65]. Items of both subscales were rated on a 5-point Likert scale (1 = *never*, 5 = *always*). Further, various scales were used to assess different kinds of social support. The social support from family and friends was measured with the two subscales of the Multidimensional Scale of Perceived Social Support [66]. Four items each measured the subscales (1 = *very strongly disagree*; 7 = *very strongly agree;* „I can talk about my problems with my family.”, α = 0.87; „My friends really try to help me.” α = 0.92). Social support from fellow students was assessed with the 4-item scale Social Support from Students ([67], 1 = *strongly disagree*; 5 = *strongly agree;* „If I can’t come to university, I can easily find someone to update me or bring me course materials.”, α = 0.88). Institutional support was measured with four self-developed items and two items from Social Support from Lecturers [67]. Participants rated items such as “I was well supported by my department. “, “My lecturers encourage me.” on a 5-point Likert scale (1 = *strongly disagree*; 5 = *strongly agree*) with a reliability of α = 0.91. In addition, students were asked to state the source of financial support (e.g., from parents, scholarship, student loan and education fund, part- or full-time job).

### Statistical Analyses

Descriptive analyses were computed to report demographic variables, psychological variables (e.g., mindfulness, self-efficacy, optimism), coping strategies (acceptance, problem-solving, social support) and prevalence of depression, anxiety and stress. Hierarchical regression analyses were conducted to explore the role of predictors on depression, anxiety and stress. Variables were entered in blocks based on theoretical considerations (see Lazarus’s transactional model of stress and coping [13] and Berry’s model of acculturative stress [11]). In Step 1, demographic variables (age, gender, graduation status, parents’ education, continent of origin, length of stay) were entered as control variables. In Step 2, psychological factors (mindfulness, optimism, self-efficacy) were added to account for internal resources. Step 3 included coping strategies (acceptance, problem-solving, and social support) to capture active management of stressful situations. Finally, acculturative stress was entered in Step 4 to examine its unique effect on mental health outcomes above and beyond psychological resources and coping strategies.

Continuous predictors were age, time spent in Germany, psychological variables and coping strategies. Categorical predictors were gender, graduation, continent of origin, parents’ education and source of financial support. Categorical variables were dummy-coded for data analysis. All analyses were performed with IBM SPSS Statistics (Version 29). A p-value of less than 0.05 was considered statistically significant.

## Results

### Preliminary analyses

Before analyzing the data, assumptions for regression analysis (including checking for outliers, multicollinearity, homoscedasticity, independence of residuals, and linearity) were tested. Regarding outliers, one outlier (> 3 *SD*) was found for depression, one for anxiety, and two for acculturative stress. Analysis with and without the outliers did not result in differences in the significance of the effects. Thus, the outliers were retained in the analyses. Given the conceptual relatedness of the included psychological variables, potential multicollinearity was carefully examined for all predictor variables included in the regression models. Multicollinearity was tested using Variance Inflation Factors (VIF), with all VIF values below 10 indicating no evidence for multicollinearity. Specifically, VIFs for the psychological variables ranged from 1.33 to 1.81, and VIFs for coping variables ranged from 1.23 to 1.58, suggesting that multicollinearity does not bias the regression estimates. Linearity was checked through scatter plots that showed a linear relationship between independent and dependent variables. Homoscedasticity was tested using the Breusch-Pagan test. The results indicated homoscedasticity for all outcome models (depression: χ^2^ = 0.35, *p* =.553; anxiety: χ^2^ = 0.98, *p* =.322; acculturative stress: χ^2^ = 3.02, *p* =.082). Independence of residuals was assessed using Durbin-Watson statistics. All values were close to 2, indicating no autocorrelation between the residuals.

### Demographic characteristics

Of the 327 international students, 201 (61.5%) were female. The mean age of the participants was 26.21 years (*SD* = 4.07). Fifty-six per cent were graduate students, 23% were undergraduate students, and 21% were postgraduate students. Most of the students were enrolled in engineering studies (38.8%, e.g., photonics, artificial intelligence, cyber security), health and social science studies (32.1%, e.g., psychology, medicine, molecular life sciences) and business studies (9.5%). The sample consisted of international students from 76 countries. 63% of the students came from Asia (29.1% India, 12% China, 10.3% Russia, 9.9% Iran), followed by Europe (19.3%; 22.2% Italy, 12.7 Ukraine, 9.5% Georgia). The average length of stay in Germany was 29.14 months (*SD* = 26.14). The students were studying at different universities mainly located in the east part of Germany (Thuringia: 69.2%, Saxony: 8.3%, Brandenburg: 6.8%). Most students had parents with an academic background (71.9%). More than half of the international students had jobs and got financial support from parents (51.1%). Only between 40.3% and 42.4% of the international students agreed that they were well supported by their faculty, department, institution or international office.

### Prevalence of mental health issues and acculturative stress

The mean score of the PHQ-9 was 10.27 (*SD* = 6.93), indicating moderate depressive symptoms. Further, 46.5% of the international students reported moderate-to-severe symptoms of depression. In addition, 31.2% (*n* = 102) of the international student sample indicated suicidal ideation or thoughts of self-harm in the last 2 weeks (PHQ-9 item 9 ≥ 1). The mean score of GAD-7 was 9.39 (*SD* = 5.50), indicating mild anxiety symptoms. However, 46.8% of the participants showed moderate-to-severe symptoms of anxiety. In addition, only 10.9% of the international students with moderate-to-severe levels of depression and/or anxiety received professional help (e.g., psychotherapy) for their mental health issues. Further, 13% (*n* = 42) had been diagnosed with a mental disorder since 2020, but only 26.1% (*n* = 11) received treatments such as psychotherapy. The most reported mental disorder was depression (73.8%, *n* = 31), followed by anxiety (45.2%, *n* = 19). 17 students who were diagnosed with depression indicated suicidal ideation or thoughts of self-harm in the last 2 weeks (PHQ-9 item 9 ≥ 1). In addition, international students experienced moderate acculturative stress. Further, 75.3% of the international students were interested in taking part in a stress management training and/or mindfulness intervention. The descriptive statistics for each investigated variable are shown in Table 1.

### Regressions on acculturative stress, depression and anxiety

Separate hierarchical regressions were performed on depression, anxiety and acculturative stress. Predictors were entered in blocks: Step 1: demographics; Step 2: psychological factors (mindfulness, optimism, self-efficacy); Step 3: coping strategies (acceptance, problem-solving, social support); Step 4: acculturative stress (for depression and anxiety models) to examine its unique contribution beyond other predictors. The results of the hierarchical regression analyses are presented in Tables 2 and 3.

**Table 2:** Results of hierarchical multiple regression analyses of depression, anxiety, and acculturative stress

	Depression		Anxiety		Acculturative Stress	
	β	*p*	β	*p*	β	*p*
Demographics						
Age	−0.016	0.779	0.004	0.937	0.122	0.058
Gender	0.091	0.052	**0.098**	**0.036**	0.029	0.591
Undergraduate	0.065	0.274	**0.119**	**0.045**	−0.070	0.307
Graduate	0.007	0.912	0.102	0.109	−0.010	0.891
Length of stay in Germany	−0.033	0.516	−0.022	0.665	0.021	0.725
North America	−0.075	0.122	**−0.148**	**0.002**	0.003	0.950
South America	−0.085	0.106	−0.079	0.133	0.058	0.337
Asia	−0.090	0.144	**−0.156**	**0.011**	**0.223**	**0.002**
Africa	−0.061	0.267	−0.103	0.058	**0.164**	**0.009**
Secondary education	−0.128	0.250	**−0.222**	**0.045**	0.134	0.297
Higher education	−0.077	0.507	−0.149	0.194	0.190	0.154
Psychological Variables						
Optimism	**−0.177**	**0.002**	**−0.121**	**0.029**	0.066	0.301
Self-efficacy	−0.093	0.430	**−0.177**	**0.003**	−0.063	0.351
Mindfulness	**−0.251**	**< 0.001**	**−0.219**	**< 0.001**	**−0.192**	**< 0.001**
Coping						
Problem solving	−0.015	0.773	0.080	0.115	−0.012	0.842
Acceptance	−0.065	0.195	**−0.115**	**0.023**	0.068	0.243
Social support with family	−0.041	0.410	−0.060	0.218	**−0.115**	**0.043**
Social support with friends	−0.072	0.189	**−0.114**	**0.036**	−0.005	0.882
Social support from fellow students	**−0.136**	**0.014**	−0.049	0.372	−0.058	0.161
Institutional support	0.068	0.192	−0.009	0.868	**−0.097**	**0.044**
Source of financial support						
Parents	−0.014	0.791	0.049	0.337	−0.036	0.679
Scholarship	0.000	0.994	0.016	0.747	−0.127	0.213
Student loan and education fund	−0.001	0.981	0.004	0.928	0.013	0.933
Job	−0.038	0.430	0.005	0.918	0.060	0.466
Acculturative stress	**0.252**	**< 0.001**	**0.208**	**< 0.001**	--	--

Male was coded as 0, female as 1; graduation: post graduate = reference group; continent of origin: Europe = reference group; education: no/primary education = reference group

**Table 3:** Hierarchical regression model changes for depression, anxiety, and acculturative stress

	Depression				Anxiety				Acculturative stress			
	*R* ^2^	*R* ^2^_adj_	*R*^2^ Change	F Change	*R* ^2^	*R* ^2^_adj_	*R*^2^ Change	F Change	*R* ^2^	*R* ^2^_adj_	*R*^2^ Change	F Change
Predictors												
Step 1: Demographics	0.043	0.010	0.043	1.29	0.079	0.047	0.079	2.47**	0.104	0.073	0.104	3.33***
Step 2: Psychological variables	0.331	0.300	0.287	44.64***	0.352	0.323	0.273	43.82***	0.175	0.138	0.071	8.89***
Step 3: Coping	0.372	0.322	0.041	1.98*	0.398	0.351	0.046	2.32*	0.229	0.168	0.055	2.14*
Step 4: Acculturative stress	0.421	0.372	0.049	25.41***	0.432	0.384	0.033	17.61***	--	--	--	--

* *p* <.05, ** *p* <.01, *** *p* <.001, R^2^_adj_ = adjusted R^2^

Hierarchical regression analyses showed that demographics accounted for only a small proportion of the variance (R² = 0.04–0.10). When psychological variables were added in Step 2, the explained variance increased substantially (ΔR² = 0.27–0.29), identifying them as the primary predictors of mental health outcomes. Coping strategies contributed a modest additional proportion of variance (ΔR² = 0.04–0.06), whereas acculturative stress accounted for a small, yet noteworthy, further increase in the depression and anxiety models (ΔR² = 0.03–0.05).

Among the psychological variables, mindfulness showed the largest standardized effects across outcomes. Higher levels of mindfulness were associated with lower levels of depression (*β* = −0.251, *p* <.001), anxiety (*β* = −0.219, *p* <.001) and acculturative stress (*β* = −0.192, *p* <.001), suggesting a small to moderate practical impact. Social support, optimism and self-efficacy were also significantly associated with outcomes. However, their effects were smaller compared to mindfulness, indicating modest contributions. Higher social support from fellow students were related to lower levels of depression (*β* = −0.136, *p* =.014), whereas higher levels of social support from friends were associated with lower levels of anxiety (*β* = −0.114, *p* =.036). In addition, higher levels of social support from family (*β* = −0.115, *p* =.043), and institutional support (*β* = −0.097, *p* =.044) were related to lower levels of acculturative stress. In addition, higher levels of optimism were negatively associated with depression (*β* = −0.177, *p* =.002) and anxiety (*β* = −0.121, *p* =.029), whereas higher levels of self-efficacy were negatively related to anxiety (*β* = −0.177, *p* =.003). Only the coping strategy acceptance was significantly negatively related to anxiety (*β* = −0.115, *p* =.023). Further, acculturative stress was a significant predictor of depression (*β* = 0.252, *p* <.001) and anxiety (*β* = 0.208, *p* <.001).

Only a few demographic variables were significant predictors for anxiety or acculturative stress (see Tables 2 and 3). In the final model, gender (*β* = 0.098, *p* =.036), graduation (undergraduate: *β* = 0.119, *p* =.045), education (secondary: *β* = −0.222, *p* =.045) and home country (North America: *β* = −0.148, *p* =.002, Asia: *β* = −0.156, *p* =.011) were significant predictors of anxiety, while home country (Asia: *β* = 0.223, *p* =.002, Africa: *β* = 0.164, *p* =.009) was a significant predictor of acculturative stress. None of the demographic variables was a significant predictor for depression. Female international students had higher levels of anxiety. Students from North America and Asia (when compared to students from Europe) demonstrated lower levels of anxiety. However, students from Asia and Africa (when compared to students from Europe) showed higher acculturative stress symptoms. Undergraduate students (when compared to postgraduate students) showed higher levels of anxiety. Students whose parents had a secondary education had lower anxiety than students whose parents had no or elementary education. Although statistically significant, the effects of demographic factors were generally small. This suggests that demographic factors explained only a limited proportion of variance in the outcomes, contributing marginally relative to psychosocial variables such as mindfulness, optimism, and social support. Thus, their overall practical influence on students’ outcomes is limited.

## Discussion

Our study examined the prevalence of mental health issues in a sample of international students in Germany. Further, we investigated the association of demographics, psychological variables (self-efficacy, optimism, mindfulness) and coping strategies with acculturative stress and mental health issues.

### Acculturative stress and mental health issues among international students

Nearly half of the international students in our study reported clinically relevant symptoms of depression and anxiety, indicating a substantially higher burden compared to domestic student populations in Germany (Prado et al. [68]: 34.9% depression in 2022; Schaller & Karing [69]: 32% depression, 29% anxiety in 2022). High rates of depression and anxiety have also been reported among international students in other European countries [7, 22]. This finding highlights international students as a particularly vulnerable group within higher education systems. The greater international students’ mental health issues seem to be related to a higher number of stressors to which these students are exposed. International students face several challenges, such as language barriers, financial concerns, and adjustments to a new culture and another academic system [3–5]. In line with this, international students in our study experienced moderate levels of acculturative stress, a finding also reported by Rajab et al. [96].

A notable finding of this study is the relatively high proportion of participants endorsing thoughts of death or self-harm on PHQ-9 item 9 (31.2%), which should be interpreted as a screening indicator rather than clinically assessed suicidal ideation. This proportion exceeds that reported for domestic students in Germany (19.2% in 2022, PHQ-9; Prado et al. [68]), but is consistent with findings from other studies with international student samples, such as Prado et al. [68] (28.2% in 2022, PHQ-9; at Leipzig University) and Bi et al. [55] (28.6% in 2021 in China, PHQ-9). According to the interpersonal theory of suicide [71, 72], suicidal desire arises from the co-occurrence of thwarted belongingness (feeling the loss of social connections and support) and perceived burdensomeness (individual perceives themselves as a burden on social groups or others). In the context of international students, these processes may be relevant given common experiences of social isolation, discrimination, and academic stress. Further, previous research has linked perceived burdensomeness more consistently to suicidal ideation in international student samples than thwarted belongingness [73, 74, 97]. Despite growing evidence on risk and protective factors, there remains a lack of suicide-specific interventions tailored to international students [75]. Existing recommendations [75] emphasize culturally sensitive services (e.g., cultural competency training for stakeholders; multicultural counselling services), early risk screening (e.g., using culturally appropriate tools; gatekeeper training), and proactive intervention strategies (e.g., information campaigns, mental health training, peer-based training). At a structural level, improving equity in access to care is essential. Policymakers and institutions are therefore encouraged to collaborate with international student bodies and local service providers to ensure accessible and appropriate mental health support [75]. Altogether, these findings suggest an important gap between identified risk indicators and targeted prevention efforts among international students.

### Protective and risk factors against acculturative stress, depression, and anxiety

From the transactional theory of stress and coping [13, 58] and the acculturative stress theory [11], personal resources are critical to the outcomes (e.g., acculturative stress, mental health issues). Our findings are in line with these theories [11, 13, 58] and previous results from studies with university students [77, 78, 93], in that higher levels of mindfulness, optimism, and self-efficacy were related to lower mental health issues and stress. However, few studies have so far investigated protective factors for international students’ mental health and existing results have been mixed [6, 21]. Notably, mindfulness emerged as the strongest protective factor across all outcomes in our study. This finding is particularly important because it supports our hypothesis that acceptance-based processes are especially adaptive in low-control contexts such as studying abroad. In particular, those that foster acceptance and non-judgmental awareness may be more effective than purely cognitive resources (e.g., optimism, self-efficacy), suggesting that mindfulness-based approaches may be especially promising from an intervention perspective. However, it should be noted that these findings are cross-sectional and therefore do not allow causal conclusions or a direct test of resilience processes. Rather, they are consistent with a resilience-oriented interpretation of protective factors in acculturative contexts. Future research should explicitly test competing theoretical mechanisms (e.g., acceptance-based versus cognitive-resource models) using longitudinal or mediation designs to better distinguish how different personal resources contribute to adaptation processes in acculturative contexts.

Another important result from our study is that acceptance was associated with lower anxiety, whereas problem-solving showed no significant relationship with mental health outcomes. Consistent with the finding by Szabo et al. [49], this further supports the adaptive role of acceptance-based coping in acculturative contexts. Our results align with the theoretical distinction between primary and secondary control strategies [47, 49]: in situations characterized by low controllability (e.g., studying abroad), acceptance-based coping may be more effective than attempts to directly change the stressor. From an intervention perspective, this suggests that interventions for international students may benefit from emphasizing secondary coping strategies (e.g., acceptance) rather than primarily focusing on primary strategies (e.g., problem-solving, active coping) in uncontrollable contexts.

Extending previous results obtained from international students [28, 33, 51], our study showed that the source of social support is essential for international students’ mental health. Different types of social support were differentially associated with specific outcomes, with social support from fellow students being linked to lower depression, support from friends to lower anxiety, and family/institutional support to lower acculturative stress. This differentiated pattern suggests that interventions should go beyond a general promotion of social support and instead target specific support networks depending on the relevant psychological outcome.

Acculturative stress was positively associated with depression and anxiety, supporting previous findings and Berry’s acculturative stress model [11, 59, 70]. This suggests that perceived inability to cope with acculturative demands represents a key risk for mental health issues among international students [14, 55]. From an intervention perspective, international students can use a range of strategies to manage acculturative stress, including seeking peer support, engaging with university-provided resources (e.g., counselling services and language courses), and participating in cultural associations. Our findings, together with previous research [28, 53, 54], suggest that peer support plays a particularly important buffering role in reducing acculturative stress, especially when it involves contact with host domestic students or locals. Accordingly, structured university programs such as peer mentoring initiatives may facilitate socialization between international students and host domestic students or locals, promoting cross-cultural understanding and acceptance [79]. In addition, cultural associations appear to provide an important space for forming co-national friendships, reducing feelings of isolation, and strengthening students’ sense of belonging within the university context [80]. These findings suggest that both host-country and co-national networks serve complementary functions in supporting international students’ adaptation. Despite their potential benefits, formal counselling services remain underutilized among international students [53, 81]. This underutilization is often linked to stigma, limited awareness, and concerns about cultural appropriateness of available services [53, 81]. This suggests that improving accessibility and cultural sensitivity of mental health services is crucial for increasing their effectiveness in supporting students facing acculturative stress.

With regard to demographic variables, only a limited number of variables (gender, graduation, education, home country) were significantly associated with acculturative stress and anxiety, and effect sizes were generally small. Consistent with previous research [94], international students from Asia and Africa reported higher levels of acculturative stress than European students. This may be explained by greater cultural similarity among European students, which may facilitate adaptation [94], whereas students from Asia and Africa may experience higher acculturative stress due to greater cultural distance and value differences (e.g., independence and individuality vs. interpersonal dependence and conformity [82, 94]). Consistent with previous studies [31], female international students reported higher levels of anxiety. This may be related to gender-specific stressors such as cultural and family expectations and traditional gender roles [31]. However, no association was found between gender and depression in the multivariate models, which is inconsistent with most previous research [32–34] and suggests that this relationship may be explained by other factors such as acculturative stress. Similarly, lower parental education and undergraduate status were associated with higher anxiety, which may reflect increased financial strain, reduced access to resources, parental expectations or transition-related stress during early stages of studying abroad [31, 83]. Overall, however, demographic effects were mostly small and less consistent than psychological predictors.

However, our findings should be interpreted in light of the study design. As all variables were assessed using self-report measures within a single survey, the observed associations may be partially influenced by shared method variance. This may have led to inflated estimates of the relationships, particularly with regard to the identified protective effects of psychological resources and social support. Thus, this should be considered when interpreting both the strength of associations and the interpretation of protective factors.

Although several predictors reached statistical significance, many effect sizes were modest (*β* = 0.10–0.25). According to Funder and Ozer [84], effect sizes should be interpreted in context rather than by rigid cutoffs, as even small effects can be meaningful in applied settings. For instance, even modest effects of mindfulness, optimism, or social support could result in practically meaningful improvements when implemented in university-wide interventions or preventive programs for international students. Future research should thus examine whether the observed effects result in clinically or practically significant improvements in international students’ mental health.

### Implications

Our findings have several implications for universities and policymakers in preventing acculturative stress and mental health issues.

1.  Institutional support: Only about 40% of our sample reported adequate support from university facilities (e.g., faculty, institution, department, international office). Universities could implement pre-arrival screening programs to identify students at risk of mental health issues and provide culturally adapted orientation programs focusing on academic practices, host-country culture, and social integration. Accessible and tailored counselling services should be offered, including digital mental health supports for students who may not seek in-person services. Peer mentoring programs can facilitate social integration and provide additional support. Several studies have already reported the positive effects of these programs on international students’ well-being and retention rates [86].
2.  Psychological interventions: Given that mindfulness showed the strongest associations with all outcomes, interventions prioritizing acceptance-based approaches (e.g., mindfulness-based programs) may be particularly effective. In contrast, interventions focusing solely on problem-solving skills may be less beneficial in the context of acculturative stress, where many stressors are not directly controllable [47, 49]. Nair and Otaki [87] recommend embedding such interventions within the curriculum. Further, using a stepped-care approach [88], students with mild symptoms could access self-guided digital interventions, while students with moderate to severe distress could receive targeted in-person counselling.
3.  Social network: Interventions should strengthen the structure of social networks rather than only increasing overall perceived support. Programs should connect students to specific networks (peers, host domestic students, institutional contacts) based on their psychological needs, considering the functional specificity of different support sources.
4.  Policy and funding: Policymakers could support these initiatives through funding and research on effective, culturally sensitive interventions and preventive measures for international students’ mental health.

### Limitations

There are several limitations that should be considered when interpreting the findings of this study: A major limitation concerns the sampling strategy, which affects the generalizability of the results. Participants were recruited using convenience and snowball sampling via mailing lists and social media. This recruitment strategy likely introduced substantial self-selection bias, potentially leading to an overrepresentation of students who are either particularly interested in mental health topics or currently experiencing psychological distress. In addition, the sample shows a strong regional clustering, with most participants recruited from universities located in eastern Germany. This regional concentration may further limit the generalizability of the findings to international students in other regions of Germany or to other national contexts. Moreover, the sample is characterized by relatively high parental education levels and a potential overrepresentation of specific academic disciplines, which may further restrict representativeness. Altogether, the reported prevalence rates and observed associations should be interpreted with considerable caution, and generalizations beyond the studied sample are limited. A second limitation is the cross-sectional design, which precludes any conclusions about causality or directionality. The identified protective and risk factors should therefore be interpreted as correlates rather than determinants of mental health outcomes. Thus, future research should conduct more longitudinal studies to investigate the relationship between possible protective and risk factors with the investigated outcome variables over time. Alternative model specifications, such as treating acculturative stress as a parallel predictor, could be explored in future research to further clarify the directional relationships between coping, stress, and mental health outcomes. Further, qualitative studies are needed to explore international students’ experiences in depth. Thus, combining a mixed-method approach with a longitudinal and multi-national study seems to be an important step toward a deeper understanding of the protective and risk factors of acculturative stress and mental health issues among international students and the development of interventions. Moreover, although several strategies were used to reduce common method bias (e.g. research reasons and instructions were explained to international students, reverse coded items were used, see also [89]), the exclusive reliance on self-report data from a single source remains a potential source of bias. This may have resulted in an overestimation of the observed associations, especially concerning the protective role of psychological resources and social support. The use of self-reported measures also increases the risk of response bias. For instance, the GAD [63] and the PHQ [61] are screening instruments and therefore only allow for probable, not clinical, diagnoses of generalized anxiety disorder and major depressive disorder [90], whereas structured clinical interviews remain the gold standard for psychiatric diagnosis [91, 92]. Altogether, future studies should consider incorporating multiple data sources to reduce common method bias and improve diagnostic validity [89]. In addition, the assessment of institutional support relied on self-developed items. Although internal consistency was strong, the lack of prior validation limits the interpretability and comparability of these findings. Future research should validate this measure or use established instruments. Finally, the interpretation of suicidal ideation assessed via item 9 of the PHQ-9 requires caution. This item is a screening indicator rather than a diagnostic assessment. Future research should use validated clinical assessments to more accurately capture suicidal ideation among international students.

## Conclusions

In conclusion, the present study found that international students in our sample showed high rates of depression and anxiety. Further, a high proportion of international students reported suicidal ideation or thoughts of self-harm. In addition, mindfulness, social support, self-efficacy, optimism and acceptance were significantly associated with lower levels of mental health symptoms and may represent potentially relevant protective factors. However, due to the cross-sectional nature of the study, these associations should not be interpreted as causal effects.

The findings underscore the importance of recognizing mental health issues among international students and highlight the potential relevance of strengthening psychological and social resources within this population. Universities may consider incorporating such factors into interventions for international students. We suggest that such interventions be embedded within the curriculum (see [87]). Additionally, ensuring access to adequate psychological services (e.g., counselling, hotlines, therapy services) and broader institutional support may be beneficial. Further longitudinal and intervention research is necessary to evaluate the efficacy of such approaches.

## Data Availability

The datasets used during the current study are available from the corresponding author on reasonable request.

## Abbreviations

GAD: Generalized Anxiety Disorder scale
HFERST: Heidelberg Form for Emotion Regulation Strategies
MAAS: Mindfulness Attention and Awareness Scale
PHQ: Patient Health Questionnaire-9 scale
VIF: Variance Inflation Factors
